# Stress-induced mast cell activation contributes to atherosclerotic plaque destabilization

**DOI:** 10.1038/s41598-019-38679-4

**Published:** 2019-02-14

**Authors:** H. Maxime Lagraauw, Anouk Wezel, Daniël van der Velden, Johan Kuiper, Ilze Bot

**Affiliations:** 1 Division of BioTherapeutics, Leiden Academic Centre for Drug Research, Leiden University, Leiden, The Netherlands; 2 Department of Surgery, Leiden University Medical Center, Leiden, The Netherlands; 3 Department of Rheumatology, Leiden University Medical Center, Leiden, The Netherlands; 4 Present Address: Boehringer Ingelheim Pharma GmbH & Co, Biberach an der Riß, Germany; 5 Present Address: Haaglanden Medical Centre, Westeinde Hospital, The Hague, The Netherlands; 6 Present Address: Hogeschool Utrecht, Utrecht, The Netherlands

## Abstract

Mast cells accumulate in the perivascular tissue during atherosclerotic plaque progression and contribute to plaque destabilization. However, the specific triggers for mast cell activation in atherosclerosis remain unresolved. We hypothesized that psychological stress-induced activation of mast cells may contribute to plaque destabilization. To investigate this, apoE^−/−^ mice on Western-type diet were exposed to 120′ restraint stress. A single episode of restraint caused a significant increase in mast cell activation in the heart. In addition to a rise in serum corticosterone and changes in circulating leukocyte populations, we observed an increase in the circulating pro-inflammatory cytokine interleukin (IL)-6 in the stressed mice. Subsequent characterization of the atherosclerotic plaques revealed a high incidence and larger size of intraplaque hemorrhages in stressed mice. In mast cell-deficient apoE^−/−^ mice, restraint stress affected circulating leukocyte levels, but did not increase plasma IL-6 levels. Furthermore, we did not observe any intraplaque hemorrhages in these mice upon stress, strongly indicating the involvement of a mast cell-dependent response to stress in atherosclerotic plaque destabilization. In conclusion, we demonstrate that acute stress activates mast cells, which induces the incidence of intraplaque hemorrhage *in vivo*, identifying acute stress as a risk factor for atherosclerotic plaque destabilization.

## Introduction

Acute cardiovascular syndromes (ACS), such as acute myocardial infarction and stroke remain principle causes of death worldwide^1,2^. In addition to traditional risk factors for ACS such as dyslipidemia, diabetes and obesity, psychological stress is receiving increased attention as both a contributing factor to various disorders, including cardiovascular disease^3^. For instance, the INTERHEART study indicated that the presence of chronic psychosocial stressors, such as work stress, increases the risk of myocardial infarction^4^, and also acute stress has been associated with an increase incidence of ACS^5^. Very recently, activity of the amygdala, an area of the brain involved in stress, was seen to associate with the incidence of ACS via bone marrow activation and arterial inflammation^6^. Although these data remain associative, the immune system seems to actively participate in stress-induced ACS. Previously, we and others have established a key role for the inflammatory mast cell in atherosclerosis and especially in the destabilization of advanced atherosclerotic plaques^7,8^. Furthermore, these experimental animal data are in line with human data, correlating perivascular mast cell numbers and activation status with disease progression and the incidence of ACS^9^. As mast cells are shown to accumulate in and near atherosclerotic lesions, these cells are uniquely located to respond to acute triggers and subsequently release their pro-atherogenic content. Being in close proximity to perivascular neurons^10^ and expressing different types of neuropeptide and hormone receptors, a neuron-mast cell connection is likely to play an exacerbating role in cardiovascular diseases. Combined, the epidemiological and experimental data suggest a pro-atherogenic role for stress-induced mast cell activation. However, the direct effects on advanced atherosclerosis, plaque stability and its implications for CVD have not yet been established. In this study we therefore evaluated mast cell-dependent effects of acute stress on atherosclerotic lesion stability in mice.

## Results

### Restraint stress activates perivascular mast cells

**Figure 1:**
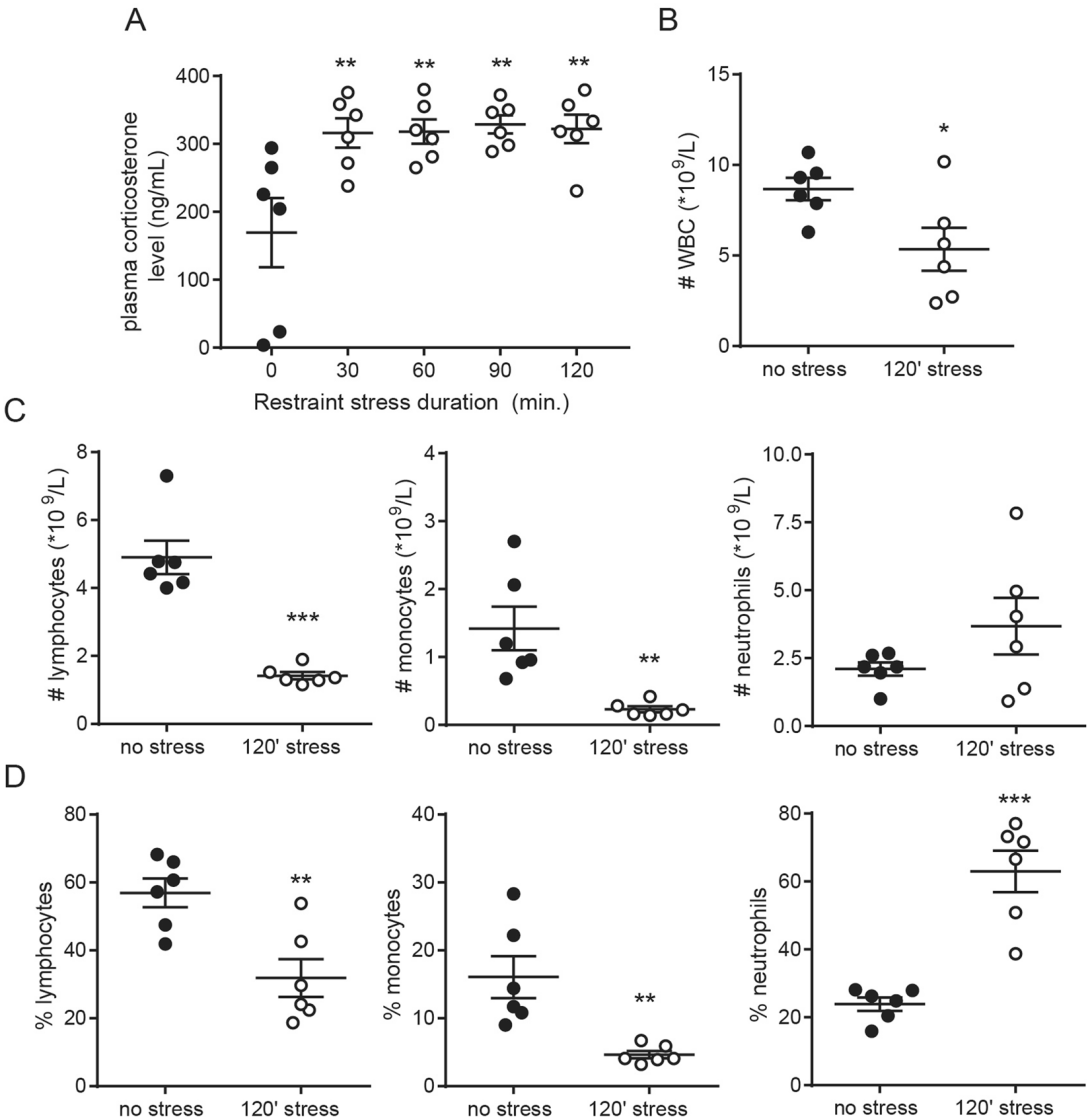
(**A**) Restraint stress significantly increased plasma corticosterone levels (n = 6 per group, one-way ANOVA followed by Dunnett’s multiple comparisons test). (**B**) Automated differential cell counting analysis of blood from non-stressed and 120′ stressed apoE^−/−^ mice showed a significant decrease in circulating WBC levels, which was mainly caused by a reduction in (**C**) lymphocytes and monocytes directly after stress exposure, both in numbers and as percentage of the total WBC population. Circulating neutrophil numbers were increased. n = 6 per group, *P < 0.05, **P < 0.01, ***P < 0.001. All data in B and C were analyzed using a two-sided unpaired student T-test.

**Figure 2:**
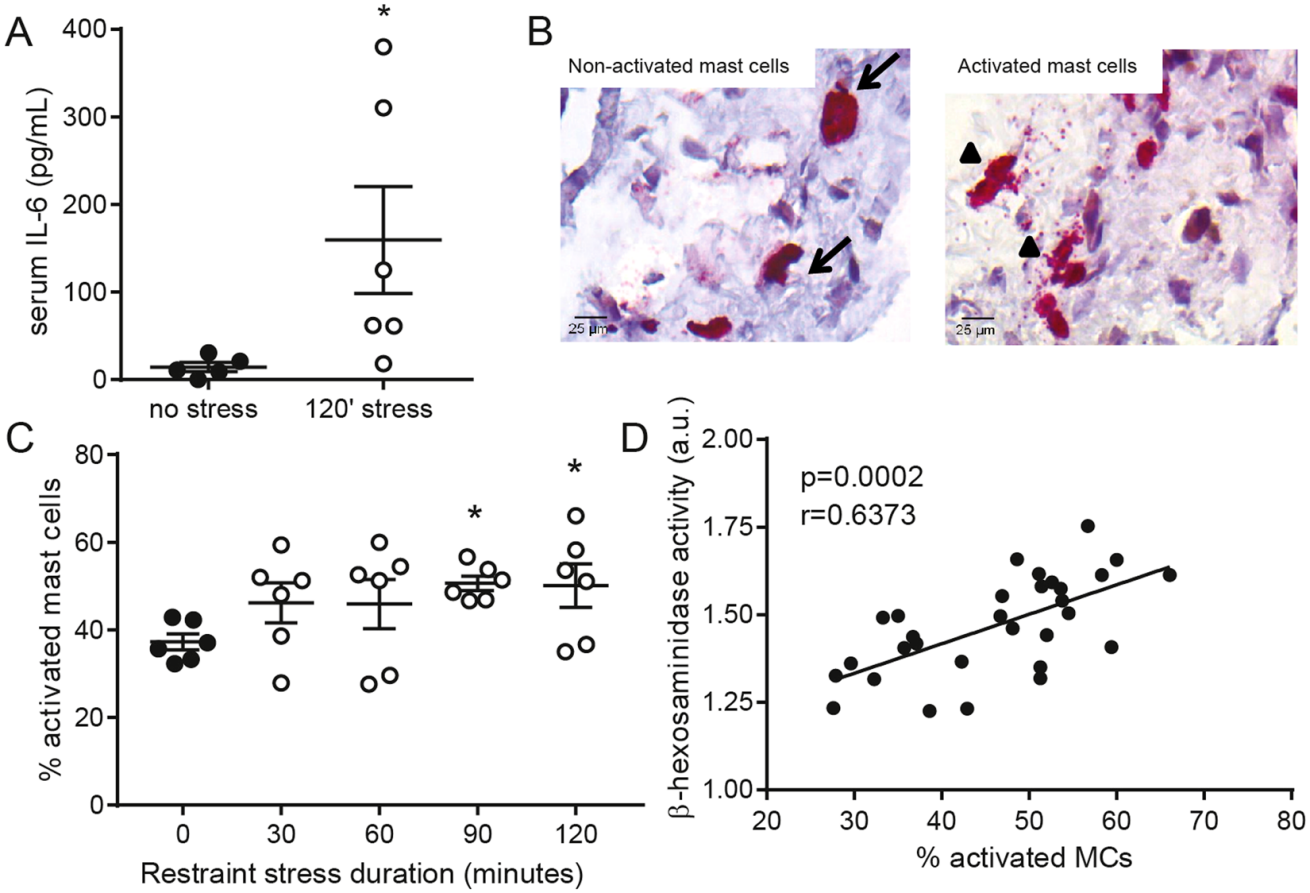
(**A**) Plasma IL-6 levels were significantly increased in stressed versus non-stressed mice (n = 6 per group, Mann-Whitney test). (**B**) Representative images of resting (non-activated, arrows) and degranulating (activated, arrow heads) perivascular mast cells in the aortic root. (**C**) Restraint stress increased mast cell activation in the aortoc root (n = 6 per group, one-way ANOVA followed by Sidak’s multiple comparisons test). (**D**) The percentage of activated aortic root mast cells significantly correlated with serum β-hexosaminidase activity levels. (Spearman’s correlation, all mice pooled, n = 30). *P < 0.05.

To evaluate the effect of acute physical and psychological stress on perivascular mast cell activation we subjected male apoE^−/−^ mice to a restraint stress protocol. A time course experiment was performed to determine the optimal stress time resulting in mast cell activation. Restraint stress induced a quick and strong increase in circulation glucocorticoid levels (P < 0.05, Fig. 1A), indicating HPA-axis activation. Furthermore, as shown before^11^, we observed a significant decrease in total white blood cell counts (Fig. 1B), which was caused by a reduction in blood monocyte and lymphocyte numbers, while the amount of circulating neutrophils was not affected by stress (Fig. 1C). The relative leukocyte composition in blood shifted to an increased percentage of neutrophils and a reduction in lymphocytes and monocytes (Fig. 1D). Serum IL-6 levels were significantly higher in the stressed mice compared to unstressed mice (Fig. 2A). Next, we assessed perivascular mast cell numbers and activation status by immunohistochemical staining of heart cross-sections at the level of the aortic root. While absolute mast cell numbers in the perivascular area of the aortoc root were similar in all groups, the percentage of activated mast cells, scored by the presence of granules deposited outside the mast cell (Fig. 2B), was significantly increased upon stress exposure (37.3 ± 1.8% unstressed vs 50.7 ± 5.0% in 120′ stressed mice, Fig. 2C, P < 0.05). Serum levels of the mast cell-granule derived mediator β-hexosaminidase significantly correlated with the percentage of activated mast cells (Fig. 2D). As apoE^−/−^ mice subjected to 2 hours of restraint stress showed a prominent increase in perivascular mast cell activation we used this experimental setup for subsequent experiments.

### Stress-induced mast cell activation in atherosclerosis

**Figure 3:**
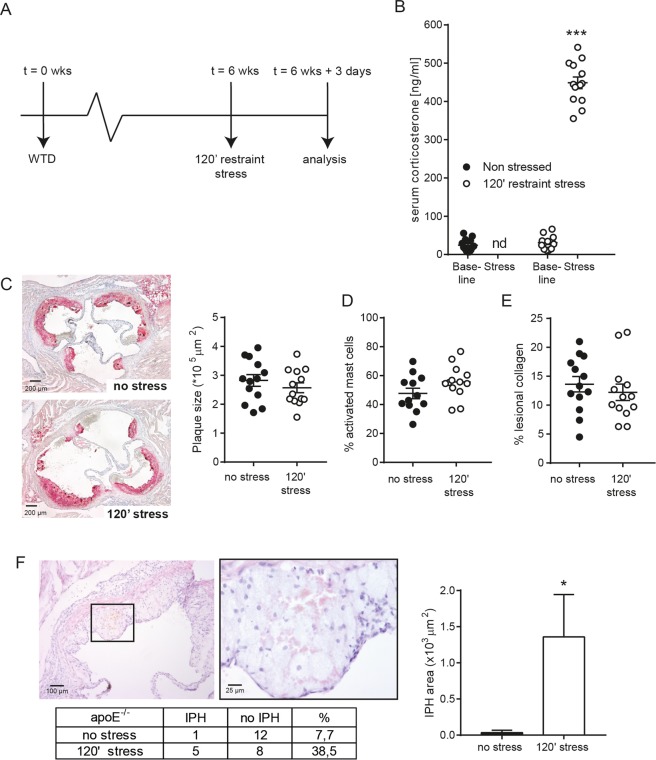
(**A**) Schematic overview of the experimental setup to evaluate the mast cell contribution to acute stress-induced cardiovascular complications. (**B**) Stress-induced increase in circulating corticosterone level in apoE^−/−^ mice (n = 13 per group, one-way ANOVA followed by Tukey’s multiple comparisons test). Atherosclerotic lesion size (**C**), mast cell activation status (**D**) or collagen content (**E**) did not differ between the groups at 3 days after the stress protocol (n = 13, two-sided unpaired student T-test). (**F**) Representative pictures of an intraplaque hemorrhage (IPH), displaying erythrocytes inside the atherosclerotic plaque. Amount and percentage of intraplaque hemorrhages in each group (Fisher’s exact test) and quantification of the IPH area demonstrates significantly larger bleedings on the stressed mice compared to the controls (Mann-Whitney test).

Next, we investigated the effects of acute stress-induced mast cell activation in an atherosclerotic setting. Western-type diet fed apoE^−/−^ mice were sacrificed three days after exposure to the stressor, a time point after which mast cell activation was been shown to have the most prominent effects on the composition of the atherosclerotic lesion^7^ (Fig. 3A). Again, 120′ restraint produced a strong stress response, indicated by a rise in glucocorticoid levels (Fig. 3B). As expected at this time-point, lesion size in the aortic root was similar between the groups (Fig. 3C). Also, at this timepoint after the stress protocol, we did not observe a significant increase in the percentage of activated perivascular mast cells in the stressed mice anymore (Fig. 3D). Apoptotic cell numbers or relative necrotic core area did not differ between stressed and unstressed mice (data not shown). Relative collagen content did not differ between the groups (Fig. 3E). Interestingly, we observed intraplaque hemorrhages (IPH), a hallmark of unstable plaques characterized by the presence of intimal erythrocytes in 5 out of 13 mice in the stress group (Fig. 3F). In contrast, only one IPH was detected in the unstressed mice. In addition, when we quantified the relative area of the intimal erythrocytes, the IPH in the stressed mice were significant larger compared to control mice (Fig. 3F).

**Figure 4:**
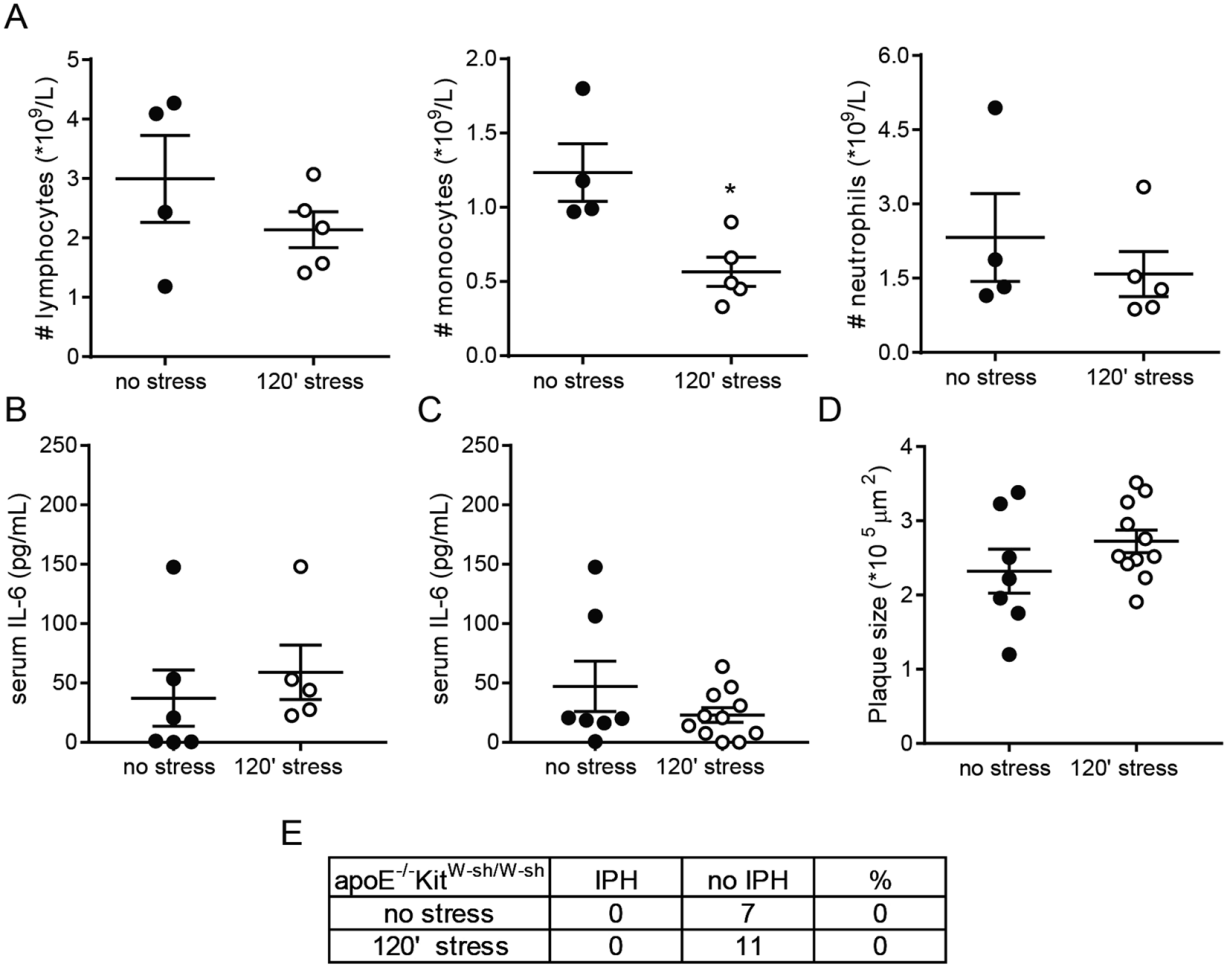
(**A**) Automated differential cell counting analysis of bloodsamples from non-stressed and 120′ stressed apoE^−/−^Kit^W-sh/W-sh^ mice (n = 4/5 per group, unpaired two-sided student T-test). (**B**) In the apoE^−/−^Kit^W-sh/W-sh^ mice, circulating IL-6 levels were not induced immediately upon stress exposure (n = 5/6 per group, unpaired two-sided student T-test), and (**C**) also after 3 days, IL-6 levels did not differ between the groups (control: n = 7, stress: n = 13 unpaired two-sided student T-test). (**D**) Plaque size in apoE^−/−^Kit^W-sh/W-sh^ was not affected at 3 days after the stress protocol (control n = 7, stress n = 11, two-tailed unpaired student T-test). (**E**) Amount and percentages of IPH in apoE^−/−^Kit^W-sh/W-sh^ mice (Fishers exact test).

To firmly establish the contribution of mast cells to the stress-dependent effects on plaque stability, we next evaluated the effects of acute stress on plaque stability in the mast cell deficient apoE^−/−^Kit^W-sh/W-sh^ mice. In this experiment, Western-type diet fed apoE^−/−^Kit^W-sh/W-sh^ mice were exposured to the stressor. A subset of mice was bled immediately after the stress protocol and as expected, stress exposure induced similar effects in the WBC populations as compared to the apoE^−/−^ mice (Fig. 4A). Strikingly however, IL-6 levels did not rise upon acute stress in the apoE^−/−^Kit^W-sh/W-sh^ mice (Fig. 4B), and also did not differ between the groups at the time of sacrifice (i.e. 3 days after the stress protocol, Fig. 4C). No significant differences in plaque size were observed between the stressed and non-stressed apoE^−/−^Kit^W-sh/W-sh^ mice (Fig. 4D), while we also did not observe a significant difference in relative collagen content of the plaques (34 ± 2% in the non-stressed group versus 31 ± 3% in the stressed group). Strikingly, no intraplaque hemorrhages could be detected in any of the aortic root plaques of either the non-stressed or stressed mice, suggesting that stress-induced plaque destabilization is, at least partly, mediated by a mast cell-dependent response (Fig. 4E).

## Discussion

Plaque inflammation and degradation of the fibrous cap are prerequisites for plaque rupture and identifying risk factors fueling the inflammatory response is key in preventing ACS^12^. The stress response and its main mediators (glucocorticoids, adrenalin and noradrenalin) are among the most potent immune modulatory agents known to men. Considerable evidence exist for a detrimental and bi-directional role for psychological stress in allergies, asthma and skin diseases^13,14^. However, such implications for ACS are less well understood. In the current study, we show that restraint stress, a mouse model for acute physical and psychological stress, activates aortic root mast cells resulting in a more unstable atherosclerotic plaque phenotype.

In the human arterial wall, mast cells have been shown to reside in the perivascular tissue^10^, but also in the intima of the human atherosclerotic plaque mast cells has been detected^15^. More specifically, mast cells were shown to accumulate in the shoulder region of coronary atheroma. There, these mast cells may degranulate and release both chymase and tryptase, as intraplaque mast cells were seen to contain both proteases^16^. These proteases, and other mast cells mediators such as chemokines and cytokines, have been shown to enhance smooth muscle cell apoptosis^17^, collagen degradation^18^, vascular leakage and overall inflammatory status of the plaque^7^, thereby potentially contributing to plaque instability. In addition, the mast cell proteases can activate matrix metalloproteinases^19^, which in turn can degrade extracellular matrix molecules, thus providing an additional mechanism via which mast cells contribute to plaque destabilization. Previous work from our group and others has demonstrated there are several neuropeptides that can induce mast cell activation in atherosclerosis, such as Substance P^20^ and Neuropeptide Y^21^. Furthermore, a recent literature review indeed showed that stress can precipitate acute coronary syndromes and demonstrated an important role for coronary mast cell activation through the stress hormone corticotropin-releasing hormone as well as other neuropeptides^22^.

Our data are in line with previous results showing cardiac mast cell degranulation and histamine secretion upon acute stress exposure^23^. We also show that restraint stress increases plasma IL-6 levels, which corresponds to observations in human studies, in which circulating IL-6 levels are associated with stress-related disorders^3^. Circulating IL-6 levels have also been shown to associate with cardiovascular disease^24,25^, and plasma IL-6 levels were actually reduced in the CANTOS trials upon treatment with canakinumab^26^. In our experimental study, IL-6 levels failed to rise upon stress exposure in the mast cell deficient mice, suggesting that the mast cell is an important source of IL-6 in episodes of stress. These data comply with a previous study, in which serum IL-6 levels were increased upon ischemia-reperfusion in wild-type mice, which did not occur in mast cells deficient W/Wv mice^27^. In our study, we measured increased mast cell activation by analyzing the amount of degranulating mast cells, however cytokines, including IL-6, can be released from mast cells via non-degranulation pathways as well. For example, IL-1 can stimulate mast cells to release IL-6 without granule release and thus active degranulation^28^.

In our study, restraint stress resulted in an increase in the amount of intraplaque hemorrhages, indicative of a more unstable plaque phenotype. Importantly, these stress-induced effects were abolished in the mast cell deficient apoE^−/−^Kit^W-sh/W-sh^, suggesting that mast cells actively participate in stress-induced intraplaque hemorrhage. In human atherosclerosis, mast cells have been shown to colocalize with intraplaque microvessels^29^ and by release of basic fibroblast growth factor, mast cells may induce the growth of these immature microvessels in the plaque^30^. The number of mast cells in carotid artery plaques also associated with the number of microvessels and interestingly, with the incidence of intraplaque hemorrhage^9^. We here speculate that focal mast cell activation may, by the release of histamine and its proteases, result in leakiness of these small and immature vessels, subsequently leading to intraplaque hemorrhage. By the deposition of erythrocytes and cholesterol these hemorrhages in turn contribute to plaque expansion and destabilization^31^.

Together, the release of mast cell mediators is likely to contribute to the induction of mast cell dependent plaque destabilization in this stress model, however this remains to be further established, also at later time points after stress exposure. The exact mechanisms via which mast cells are activated during episodes of stress remain to be elucidated as well.

In conclusion, we here demonstrate that a single episode of restraint stress, is sufficient to significantly activate perivascular mast cells, resulting in an increase in serum IL-6 and β-hexosaminidase levels. Such acute stress-induced mast cell activation in mice with established atherosclerotic lesions negatively affected plaque stability by inducing the intraplaque hemorrhage incidence. Combined these results provide further evidence for the important role of mast cells in modulating atherosclerotic plaque stability and highlight the acute stress response as a ACS risk factor.

## Methods

### Animals

All animal work was approved by the Leiden University Animal Ethics committee and performed in compliance with the Dutch government guidelines and the Directive 2010/63/EU of the European Parliament. 10–12 weeks old male apoE^−/−^ mice and mast cell deficient apoE^−/−^Kit^W-sh/W-sh^ were obtained from the local animal breeding facility and all animals had access to food and water ad libitum.

### Restraint-stress model

All stress experiments were performed between 9:00 AM and noon, a period in which individual differences in corticosterone levels in mice are relatively small^11^. 24 male apoE^−/−^ mice were subjected to restrained stress by immobilization in a well-ventilated 50 mL Corning tube for 30′, 60′, 90′ or 120′ (n = 6 per time point) while control mice were left undisturbed in their home cage. Directly after the indicated stress-time the mice were terminally anaesthetized by subcutaneous injections with ketamine (100 mg/mL), sedazine (25 mg/mL) and atropine (0.5 mg/mL). Deep anesthesia was established by toe pinch reflex, after which the mice were sacrificed. Total cell count, neutrophils, monocyte and lymphocyte counts in blood were analyzed using an automated XT-2000iV veterinary hematology analyzer (Sysmex Europe GMBH, Norderstedt, Germany). Serum IL-6 levels were determined by ELISA according to the manufacturer’s protocol (BD Biosciences). Hearts were excised and briefly fixed in 3.7% neutral-buffered formalin (Formal-Fixx; Shandon Scientific Ltd, UK) before further processing.

### Serum analysis

Blood samples for basal corticosterone analysis were drawn by tail cut between 9:00 AM and noon. Levels of corticosterone were determined using a 125-I radioimmunoassay (RIA) with a lower detection limit of 5 ng/ml, according to the manufacturer’s specifications (MP Biomedicals, Illkirch-Graffenstaden, France). To measure β-hexosaminidase levels 50 μL of serum was added to 50 μL 2 mM 4-nitrophenyl N-acetyl-b-D-glucosaminide (Sigma, Germany) in 0.2 M citrate (pH 4.5) and incubated at 37 °C for 2 hours. After addition of 150 μL 1 M Tris (pH 9.0), absorbance was measured at 405 nm.

### Atherosclerosis

Atherosclerotic lesion formation was induced by feeding male apoE^−/−^ mice (n = 39) a Western-type diet (0.25% cholesterol and 15% cocoa butter; Special Diet Services, Witham, Essex, UK) for 6 weeks. Blood samples were taken at the start of the experiment, at 2 and 5 weeks of WTD feeding by tail cut and plasma was obtained by centrifugation at 8,000 rpm for 10 min. Levels of total plasma cholesterol were measured spectrophotometrically using enzymatic procedures (Roche Diagnostics, Almere, the Netherlands). At 6 weeks mice were randomly assigned to either the undisturbed control or the 120′ restraint stress (n = 13 per group) and subjected to the stress protocol. Three days after stress all mice were terminally anaesthetized by subcutaneous injections with ketamine (100 mg/mL), sedazine (25 mg/mL) and atropine (0.5 mg/mL). Deep anesthesia was established by toe pinch reflex, after which their vascular system was perfused with PBS at a continuous low flow via heart puncture in the left ventricle. Blood was processed for Sysmex or ELISA/RIA as described above. Heart, skin and lung tissue were fixated in 3.7% neutral-buffered formalin (Formal-fixx; Shandon Scientific Ltd, UK) before further processing. A similar experimental setting was applied to evaluate the effects of stress in mast cell deficient male apoE^−/−^Kit^W-sh/W-sh^ mice (no stress: n = 7, 120′ stress: n = 13).

### Plaque morphometry and immunohistochemistry

Harvested hearts were fixated in 3.7% neutral-buffered formalin solution (Formal-fixx; Shandon Scientific Ltd, UK) and embedded in O.C.T compound (Tissue-Tek, Sakura Finetek, CA, USA). Once the aortic root was identified by the appearance of aortic valve leaflets, transverse 10-µm sections were prepared on a Leica CM 3050 S Cryostat (Leica Instruments, Nassloch, Germany) and mounted on gelatin-coated slides. Mean lesion area (in µm^2^) was calculated from six Oil-Red-O stained sections in distal direction starting at the point where all three aortic valve leaflets first appeared. Leica Qwin image analysis software was used for morphometric analysis of the atherosclerotic burden. Mast cells were visualized by chloroacetate esterase staining according to manufacturer’s protocol (CAE, Sigma, Germany) and degranulation status was assessed manually by bright-field microscopy as described previously^7^. Mast cells were identified and counted in the perivascular tissue of the aortic root at the site of atherosclerosis. A mast cell was considered resting when all granula were maintained inside the cell, while mast cells were assessed as activated when granula were deposited in the tissue surrounding the mast cell.

In addition, immunohistochemical stainings were performed to assess plaque composition and stability. Sections were stained for collagen (picrosirius red) and macrophages (MOMA-2) as described previously^32^. Necrotic core area was defined as the a-cellular, debris-rich plaque area and represented as percentage of total plaque area. Apoptosis was visualized using a terminal dUTP nick-end labelling (TUNEL) kit (Roche Diagnostics). As another measure of lesion destabilization the amount and surface area of intraplaque hemorrhages (IPH) were determined as described before^7^. Presence of masses of intimal erythrocytes, free in the plaque matrix or filling the necrotic core, was classified as intraplaque hemorrhage^33^. For detection of intraplaque hemorrhage, we analyzed all hematoxylin & eosin-stained sections (at 50 µM intervals) for each mouse having atherosclerotic plaques. Furthermore, erythrocytes were visualized by fluorescence microscopy (erythrocyte autofluorescence at 560 nm emission wavelength). All morphometric analyses were performed by blinded independent operators (I.B. and H.M.L.).

### Statistical analysis

Data are expressed as mean ± SEM. A two-tailed student T-test was used to compare normally distributed data between individual groups, while non-parametric data were analyzed using a Mann-Whitney test. For comparison of three or more different groups, data were analyzed with a one-way ANOVA followed by a Tukey’s, Dunnett’s or Sidak’s multiple comparisons test. Correlations were analyzed using a Spearman’s correlation test. Frequency data analysis was performed by means of the Fisher’s exact test. Probability values of *P* < 0.05 were considered significant.

## Data Availability

The data generated during and/or analysed in this study are available from the corresponding author on reasonable request.
